# Evaluation of the clinical relevance of the Biofire^©^ FilmArray pneumonia panel among hospitalized patients

**DOI:** 10.1007/s15010-023-02080-1

**Published:** 2023-08-12

**Authors:** Kirstine K. Søgaard, Vladimira Hinic, Daniel Goldenberger, Alexander Gensch, Michael Schweitzer, Veronika Bättig, Martin Siegemund, Stefano Bassetti, Roland Bingisser, Michael Tamm, Manuel Battegay, Maja Weisser, Daiana Stolz, Nina Khanna, Adrian Egli

**Affiliations:** 1 Clinical Bacteriology and Mycology, University Hospital Basel, 4031 Basel, Switzerland; 2 Department of Biomedicine, Applied Microbiology Research, University of Basel, Basel, Switzerland; 3 Department of Clinical Microbiology, Aalborg University Hospital, Aalborg, Denmark; 4 Institute of Medical Microbiology, University of Zurich, Gloriastrasse 28/30 8006, Zurich, Switzerland; 5 Division of Infectious Diseases and Hospital Epidemiology, University Hospital Basel and University of Basel, Basel, Switzerland; 6 Intensive Care Medicine, University Hospital Basel, Basel, Switzerland; 7 Internal Medicine, University Hospital Basel, Basel, Switzerland; 8 Emergency Medicine, University Hospital Basel, Basel, Switzerland; 9 Pneumology, University Hospital Basel, Basel, Switzerland

**Keywords:** Biofire FilmArray, Panel PCR, Pneumonia, Respiratory tract, Diagnostics, Culture

## Abstract

**Purpose:**

Panel PCR tests provide rapid pathogen identification. However, their diagnostic performance is unclear. We assessed the performance of the Biofire^©^ FilmArray pneumonia (PN)-panel against standard culture in broncho-alveolar lavage (BAL) samples.

**Methods:**

Setting: University Hospital Basel (February 2019 to July 2020), including hospitalized patients with a BAL (± pneumonia). We determined sensitivity and specificity of the PN-panel against standard culture. Using univariate logistic regression, we calculated odds ratios (OR) for pneumonia according to PN-panel and culture status, stratifying by chronic pulmonary disease. We calculated ORs for pneumonia for different pathogens to estimate the clinical relevance.

**Results:**

We included 840 adult patients, 60% were males, median age was 68 years, 35% had chronic pulmonary disease, 21% had pneumonia, and 36% had recent antibiotic use. In 1078 BAL samples, bacterial pathogens were detected in 36% and 16% with PN-panel and culture, respectively. The overall sensitivity and specificity of the PN-panel was high, whereas the positive predictive value was low. The OR of pneumonia was 1.1 (95% CI 0.7–1.6) for PN-panel-positive only; 2.6 (95% CI 1.3–5.3) for culture-positive only, and 1.6 (95% CI 1.0–2.4) for PN-panel and culture-positive. The detection rate of *Haemophilus influenzae*, *Staphylococcus aureus,* and *Moraxella catarrhalis* in the PN-panel was high but not associated with pneumonia.

**Conclusion:**

While sensitivity and specificity of PN-panel are high compared to culture, pathogen detection did not correlate well with a pneumonia diagnosis. Patients with culture-positive BAL had the highest OR for pneumonia—thus the impact of the PN-panel on clinical management needs further evaluation in randomized controlled trials.

**Supplementary Information:**

The online version contains supplementary material available at 10.1007/s15010-023-02080-1.

## Introduction

Pneumonia is a common, potentially severe infection associated with substantial morbidity and mortality. Early diagnosis of the causing microorganism and targeted antibiotic treatment is crucial especially in hospitalized patients for an improved clinical outcome [1]. Currently, the time from collection of a respiratory sample to the identification of bacterial species and the determination of antibiotic susceptibility by standard of care culture-based diagnostic methods is at least 48 h.

The Biofire FilmArray^©^ Pneumonia Panel (PN-panel) allows direct identification of pathogens from respiratory samples without the need of subculture to reach single bacterial colonies. Therefore, time to results are substantially shorter. The assay can identify the 18 most common Gram-positive and Gram-negative bacteria, nine most common viral agents causing pneumonia [2–4], and several important resistance genes such as CTX-M linked to extended spectrum beta-lactamases (ESBL), mecA/C linked to Methicillin-resistant *Staphylococcus aureus*, and carbapenemases (Table S1) [2].

However, knowledge is scarce on how well the results from this panel PCR correspond to (i) the culture-based standard-of-care results and (ii) the clinical diagnosis of pneumonia. Detection of DNA in respiratory material is not necessarily linked to replicating bacteria causing an infection and may result in an overuse of antibiotics [2]. In addition, the PN-panel is significantly more expensive compared to culture-based diagnostics. Therefore, understanding the diagnostic performance of the PN-panel is a critical step before implementing this technology in routine diagnostic workflows. Prior studies validated the assay only against standard culture but did not consider important clinical information, e.g., diagnosis of pneumonia, chronic pulmonary disease, pre-treatment with antibiotics. Thus, the relevance of PN-panel results in the clinic setting was not analysed [3, 4].

We aimed to determine the microbiological performance of the PN-panel compared to culture-based diagnostics in patients receiving a BAL, comparing findings among hospitalized patients with and without pneumonia.

## Material and methods

We performed a retrospective observational study comparing diagnostic methods of lower respiratory tract infection within hospitalized patients. The study included patients above 18 years of age who received a bronchial alveolar lavage (BAL) performed by the divisions of pulmonology and intensive care medicine at the University Hospital Basel, examined at the Division of Clinical Bacteriology and Mycology, University Hospital Basel between February 2019 and July 2020. Indications for BAL were either diagnostics of interstitial lung disease or diagnosis of acute or chronic infection. The BALs were mainly performed in sedation, using protection to avoid contamination, following the standard guidelines for bronchoalveolar lavage as previously described [5].

We identified 1078 bronchoalveolar lavage (BAL) samples analysed by both culture (standard of care and reference standard) and Biofire FilmArray^©^ Pneumonia (PN) panel (bioMérieux, Lyon, France). The study was approved by the local ethical committee (EKNZ Nr 2021-00007).

### Patient data

We extracted clinical data from the Hospital’s Clinical Data Warehouse (CDWH). Clinical data included age, gender, diagnosis of pneumonia (as documented by treating physicians using International Classification of Diseases 10th version (ICD-10) diagnosis codes), chronic pulmonary disease (e.g., chronic pulmonary obstructive disease, emphysema, chronic bronchitis and cystic fibrosis), imaging examinations (± 72 h within time of BAL). From the chart, we retrieved information on the first measurement (± 72 h within time of BAL) of body temperature (fever defined as tympanal temperature ≥ 38.0 °C), oxygen saturation as SpO_2_ in %, and laboratory measurements such as C-reactive protein (CRP) in mg/L and neutrophil leucocyte count in 10^9^/L from blood. Data completeness for these parameters was 97%. We also extracted information on recent antibiotic treatment (< 72 h), whether patients were hospitalized on intensive care unit, total length-of hospitalization stay, and in-hospital all-cause mortality.

### Microbiological data

In brief, we analysed all samples within the routine workflow for species identification of bacteria and molds and performed antibiotic resistance profiling. Presence of normal respiratory flora was noted. We performed the PN-panel according to the manufacturer’s instruction. As the PN-panel was newly implemented and the clinical relevance of findings uncertain, only viral and atypical bacterial pneumonia pathogens, were reported to the clinicians. In case of detection of an AMR gene, we informed the treating physician. Results of the cultured microorganism and susceptibility testing, as well as PN-panel results were collected (see the supplemental description in the Appendix).

### Statistical analysis

We described the patient’s data as frequency and percentage for qualitative parameters and as median and interquartile range for quantitative parameters. We compared the identification of pathogens and resistance from the PN-panel to culture-based diagnostics. We calculated sensitivity, specificity, and positive and negative predictive value (PPV, NPV) for the PN-panel using culture as the reference standard. Additionally, we examined the correlation of the semi-quantification of the PCR to the quantification in culture. Using univariate logistic regression, we calculated odds ratios (OR) with 95% confidence intervals (CI) or pneumonia according to PN-panel and culture status (PN-panel-positive only, culture-positive only, and PN-panel and culture positive), stratifying by chronic pulmonary disease. For pathogens detected in five events of pneumonia or more, we calculated OR with 95% CI for an association with pneumonia. We used binomial exact to calculate 95% CI. In the regression models, the viral pathogens were not considered.

## Results

### Clinical data and patient characteristics

During an 18-month period, we identified 840 hospitalized patients who had one or more BAL performed and examined by culture and the PN-panel (totalling 1078 samples). Table 1 summarizes the patient characteristics among patient with and without a pneumonia diagnosis. A total of 507 (60%) patients were male, the median age was 68 years (IQR 56–76 years), and 293 (35%) had chronic pulmonary disease. Median length of stay in the hospital was 6 days (IQR 3–16), 584 (70%) had either chest computed tomography scan or chest x-ray performed ± 72 h within time of BAL performance, 175 (21%) had a discharge diagnosis of pneumonia, and 144 (17%) required intensive care treatment. Among the patients with pneumonia, imaging was performed in 83%, median length of hospital stay was 12 days (IQR 7–21), 29% required intensive care treatment, and 9% died during the hospital stay (Table 1).

**Table 1:** Characteristics of 840 unique patients with one or more BAL samples examined by culture-based methods and Biofire FilmArray^*©*^ Pneumonia panel

	All patients *N* = 840 (100%)	With pneumonia^a^ *n* = 175 (21%)	Without pneumonia *n* = 665 (79%)
Males, *n* (%)	507 (60)	114 (65)	393 (59)
Females, *n* (%)	333 (40)	61 (35)	272 (41)
Age in years, median (IQR)	68 (56–76)	69 (58–77)	67 (56–75)
Chronic pulmonary disease, *n* (%)	293 (35)	51 (29)	242 (36)
Vital parameters (first measurement)			
Temperature ≥ 38 °C	40 (5)	19 (11)	31 (5)
CRP mg/L, median (IQR)	22 (3–87)	87 (32–157)	12 (2–61)
Neutrophile leucocyte count 10^9^/L, median (IQR)	5.5 (3.8–8.1)	7.4 (4.8–10.2)	5.1 (3.7–7.5)
SpO_2_, median (IQR)	95 (93–96)	94 (92–96)	95 (93–96)
Imaging ± 72 h	584 (70)	146 (83)	438 (66)
Antibiotics < 72 h before sampling	305 (36)	121 (69)	184 (28)
Length of stay in days, median (IQR)	6 (3–16)	12 (7–21)	4 (3–14)
Intensive care treatment, *n* (%)	144 (17)	51 (29)	93 (14)
In-hospital mortality, *n* (%)	36 (4)	15 (9)	21 (3)

^a^Patients with a discharge diagnosis of pneumonia (registered using ICD-10)

Among the 175 patients diagnosed with pneumonia, 19 (11%) had fever at first measurement, median CRP was 87 mg/L (IQR 32–157), neutrophil leucocyte count was 7.4 × 10^9^/L (IQR 4.8–10.2), and median oxygen saturation was 94% (IQR 92–96%).

#### Antibiotic treatment

Overall, 390 (46%) patients were treated with antibiotics (± 72 h within time of BAL). Among these patients, 305 (78%) received antibiotic treatment before the BAL was performed, whereas 85 (22%) had no antibiotics before sampling but were treated with antibiotics after the BAL. The most used antibiotics were amoxicillin-clavulanic acid (33%), piperacillin-tazobactam (46%), meropenem (14%), clarithromycin (11%), cefepime (9%), and ceftriaxone (8%).

### Microbiological test performance

#### Pathogens detected with the PN-panel and culture

Among the 840 patients, a total of 1078 BAL samples were examined by both PN-panel and culture. The PN-panel detected bacterial pathogens in 506 (47%) samples, whereas growth of these same pathogens was reported in only 185 (17%) samples after culture. The most common bacterial pathogens detected by the PN-panel were *Haemophilus influenzae* (143/1078, 13.3%), *Staphylococcus aureus* (98/1078, 9.1%), *Pseudomonas aeruginosa*, (54/1078, 5.0%), *Streptococcus pneumoniae* (54/1078, 4.0%), *Escherichia coli* (43/1078, 4.0%), *Moraxella catarrhalis* (28/1078, 2.6%), *Streptococcus agalactiae* (19/1078, 1.8%), and *Klebsiella pneumoniae* group (19/1078, 1.8%). The most common pathogens detected by culture were *S. aureus* (48/1078, 4.5%), *P. aeruginosa* (39/1078, 3.6%), *S. pneumoniae* (23/1078, 2.1%), *E. coli* (21/1078, 1.9%), and *H. influenzae* (17/1078, 1.6%) (Table 2**)**.

**Table 2:** Summary of detected pathogens in the 1078 bronchoalveolar lavages with Biofire FilmArray^©^ pneumonia panel (PN) and/or culture among 840 unique patients

Species	Pneumonia (232 BALs from 175 patients), *n*				No pneumonia (846 BALs from 665 patients), *n*			
	PN + Culture +	PN + Culture–	PN–Culture +	Total	PN + Culture +	PN + Culture–	PN–Culture +	Total
*Acinetobacter calcoaceticus-baumannii* complex	0	1	0	1	0	2	0	2
*Enterobacter cloacae* complex	4	1	0	5	4	7	0	11
*Escherichia coli*	8	4	1	13	11	20	1	32
*Haemophilus influenzae*	4	22	2	28	11	106	0	117
*Klebsiella aerogenes*	0	1	2	3	0	0	0	0
*Klebsiella oxytoca*	0	0	0	0	1	2	0	3
*Klebsiella pneumoniae* group	1	3	1	5	7	8	1	16
*Moraxella catarrhalis*	1	4	0	5	3	20	0	23
*Proteus* spp.	2	4	0	6	5	0	0	5
*Pseudomonas aeruginosa*	13	8	1	22	23	10	2	35
*Serratia marcescens*	1	1	0	2	2	5	0	7
*Staphylococcus aureus*	10	9	1	20	33	46	4	83
*Streptococcus agalactiae*	1	4	0	5	1	13	0	14
*Streptococcus pneumoniae*	4	10	1	15	16	24	2	42
*Streptococcus pyogenes*	0	2	0	2	0	3	0	3
*Total, n*	49	74	9	122	117	266	10	393

+  Positive, – Negative

When comparing results from the PN-panel to those of culture, we found that sensitivity and specificity generally were high for the pathogens covered by the PN-panel, whereas the PPVs were low for many pathogens. For *H. influenzae, M. catarrhalis, K. pneumoniae-group, Proteus *spp.,* S. pneumoniae, and S. agalactiae* the PPVs ranged between 15 and 47% (Table 3**)**.

**Table 3:** Sensitivity, specificity, positive predictive value, and negative predictive value of Biofire FilmArray^©^ pneumonia (PN)-panel compared with culture among hospitalized patients with and without pneumonia

	Pneumonia (232 BALs from 175 patients)				No pneumonia (846 BAL among 665 patients)			
	Sensitivity % (95% CI)	Specificity % (95% CI)	PPV % (95% CI)	NPV % (95% CI)	Sensitivity % (95% CI)	Specificity % (95% CI)	PPV % (95% CI)	NPV % (95% CI)
Bacterial species								
*A. calcoaceticus-baumannii* complex	–	100 (98–100)	–	100 (98–100*)	–	100 (99–100)	–	100 (100–100*)
*E. cloacae* complex	100 (40–100*)	100 (98–100)	80 (28–99)	100 (98–100*)	100 (40–100*)	99 (98–100)	36 (11–69)	100 (100–100*)
*E. coli*	89 (52–100)	98 (95–100)	67 (35–90)	100 (97–100)	92 (62–100)	98 (96–99)	35 (19–55)	100 (99–100)
*H. influenzae*	67 (22–96)	90 (86–94)	15 (4–35)	99 (97–100)	100 (72–100*)	87 (85–89)	9 (5–16)	100 (99–100*)
*K. aerogenes*	–	100 (98–100)	–	99 (97–100)	–	100 (100–100*)	–	100 (100–100*)
*K. oxytoca*	–	100 (98–100*)	–	100 (98–100*)	–	100 (99–100)	–	100 (100–100*)
*K. pneumoniae-*group	–	99 (96–100)	25 (1–81)	100 (98–100)	88 (47–100)	99 (98–100)	47 (21–73)	100 (99–100)
*M. catarrhalis*	100 (3–100*)	98 (96–100)	20 (1–72)	100 (98–100*)	100 (29–100*)	98 (96–99)	13 (3–34)	100 (100–100*)
*Proteus* spp.	100 (16–100*)	98 (96–100)	33 (4–78)	100 (98–100*)	100 (48–100*)	100 (100–100*)	100 (48–100*)	100 (100–100*)
*P. aeruginosa*	93 (66–100)	96 (93–98)	62 (38–82)	100 (97–100)	92 (74–99)	99 (98–99)	70 (51–84)	100 (99–100)
*S. marcescens*	–	100 (98–100)	–	100 (98–100*)	100 (16–100*)	99 (99–100)	29 (4–71)	100 (100–100*)
*S. aureus*	91 (59–100)	96 (92–98)	53 (29–76)	99 (97–100)	89 (75–97)	94 (92–96)	42 (31–53)	99 (99–100)
*S. agalactiae*	100 (3–100*)	98 (95–99)	20 (1–72)	100 (98–100*)	100 (3–100*)	98 (97–99)	7 (0–34)	100 (100–100*)
*S. pneumoniae*	80 (28–100)	96 (92–98)	29 (8–58)	100 (97–100)	89 (65–99)	97 (96–98)	40 (25–57)	100 (99–100)
*S. pyogenes*	–	99 (97–100)	–	100 (98–100*)	–	100 (99–100)	–	100 (100–100*)

For pathogens with less than 5 identified in total (by culture or PN panel), and for samples without culture detected pathogens, sensitivity and PPV were not calculated

*CI* confidence intervals

*95% CI were calculated using binomial exact

*When the upper limit of CI was above 100%, one-sided 97.5% CI was used

In 848/1078 (79%) of all samples, normal respiratory flora was identified by the culture-based method. In total, PN-panel detected additional 346 bacterial (including 6 atypical pneumonia bacteria and 153 viral pathogens) among 114 patients, whereas culture detected 19 additional bacteria (included in the PN panel, but not detected) and 171 other bacteria or fungi which could represent pathogens or colonizing flora (not included in the PN panel). Supplemental Table S2 lists all additional microorganisms detected in the 1078 BALs.

#### Semi-quantitative results between the PN-panel and culture

Supplemental Table S3 displays the semi-quantitative results for all 1078 samples with and without pneumonia, respectively. Of the 506 detected pathogens by PN panel, 213 (42%) had 10^4^ genome copies/mL, 146 (29%) had 10^5^ copies/mL, 76 (15%) had 10^6^ copies /mL, and 71 (15%) had >  = 10^7^ copies/mL. Of note, the proportion of *H. influenza, M. catarrhalis* and *S. aureus* in 10^4^ genome copies/mL was high among patients without pneumonia.

#### Resistance genes

Among our patients, prevalence of multi-resistant bacteria was low. In total, the PN-panel detected five CTX-M and six mecA/C and MREJ. Of these 11 resistant bacteria, only two ESBL-producing Enterobacterales and three MRSA were detected using culture-based methods (which alone did not detect additional resistant bacteria).

### Performance in the context of clinical parameters

#### Pathogens in hospitalized patients with and without pneumonia

Among the 840 hospitalized patients, one or more bacterial pathogens was detected by the PN-panel in 295 (35%) of patients (when restricting to one sample per patient). Among 175 patients with pneumonia, 72 (41%) had one or more bacterial pathogen detected by the PN-panel; 51 (29%) patients had one pathogen detected, 12 (7%) patients had two pathogens detected, and 9 (5%) patients had three or more pathogens detected. Among 665 patients without pneumonia, the corresponding detection rates were 34%, 24%, 7%, and 3% (Table 4). In culture-based methods, 20% of patients with pneumonia had a positive culture and 12% without pneumonia had a positive culture (with bacteria covered by the PN-panel). However, in the culture-based method normal respiratory flora was detected in the majority of samples, in 64% among those with pneumonia, and 83% among those without pneumonia. Of note, in 19% of patients with pneumonia and 12% without pneumonia one or more respiratory virus was detected.

**Table 4:** Number of detected pathogens among 840 hospitalized patients with and without pneumonia

	All inpatients *n* = 840 (100%)	Patients with pneumonia *n* = 175 (21%)	Patients without pneumonia *n* = 665 (79%)
	*n* (%)	*n* (%)	*n* (%)
PN-panel, any pathogen			
0	480 (57%)	85 (49%)	392 (59%)
1	238 (28%)	56 (32%)	182 (28%)
2	85 (10%)	24 (14%)	61 (9%)
≥ 3	37 (4%)	10 (6%)	28 (4%)
PN-panel, bacteria			
0	545 (65%)	103 (59%)	442 (66%)
1	208 (25%)	51 (29%)	157 (24%)
2	60 (7%)	12 (7%)	48 (7%)
≥ 3	27 (3%)	9 (5%)	18 (3%)
PN-panel, virus			
0	726 (86%)	141 (81%)	585 (88%)
1	111 (13%)	33 (19%)	78 (12%)
2	< 5	< 5	< 5
≥ 3	< 5	< 5	< 5
Culture detected bacteria			
0	723 (86%)	140 (80%)	583 (88%)
1	96 (11%)	30 (17%)	66 (10%)
2	18 (2%)	< 5	13 (2%)
≥ 3	< 3	< 5	< 5
Normal respiratory flora^a^	665 (79%)	112 (64%)	553 (83%)

^a^Normal respiratory flora e.g., *S. mitis* and other viridans streptococci, apathogenic *Neisseria* spp., *Corynebacteria* spp., *Lactobacillus* spp. and *Candida* spp

In the 232 BALs from the 175 patients with pneumonia, the most frequent detected pathogens by PN-panel included *H. influenzae* (*n* = 26)*, P. aeruginosa* (*n* = 21)*, S. aureus* (*n* = 19)*, S. pneumoniae* (*n* = 14), and *E. coli* (*n* = 12) (Table 2**)**. Detection of atypical pneumonia pathogens and viral pathogens are described in the supplemental Appendix.

The most frequently detected pathogens by culture among patients with pneumonia included *P. aeruginosa* (*n* = 14)*, S. aureus* (*n* = 11)*, E. coli* (*n* = 9), *H. influenzae* (*n* = 6)*,* and *S. pneumoniae* (*n* = 5) (Table 2 and Table S3). The culture-based method detected *Stenotrophomonas maltophilia and* Enterobacterales not covered by the PN-panel in four and seven patients, respectively*.* By the culture-based method, we also identified *Aspergillus fumigatus* (*n* = 4), *Rhizomucor* spp. (*n* = 1), *Geotrichum* spp. (*n* = 1), and unspecified mould (*n* = 5) which may cause invasive infections in immunocompromised patients (Table S4).

#### Association between detection of pathogens and pneumonia

We calculated odds ratios for pneumonia according to PN-panel and culture status (PN-panel-positive, PN-panel-positive only, culture-positive, culture-positive only, and PN-panel and culture positive), stratifying by chronic pulmonary disease. We found the strongest association for “culture-positive only” with an OR of 2.6 (95% CI 1.3–5.3). “PN-panel positive only” were not associated with pneumonia (Table 5). When stratifying by underlying chronic pulmonary disease, we found that a positive PN-panel was a predictor of pneumonia only in patients without chronic pulmonary disease, whereas a positive culture was a predictor of pneumonia in patients both with and without chronic pulmonary disease (Table 5).

**Table 5:** Crude odds ratios of bacterial pneumonia among 840 unique hospitalized patients including 175 with pneumonia, according to microbiological test results and presence of chronic pulmonary disease

	Odds ratio (95% CI)
PN-panel positive	1.4 (1.0–1.9)
Chronic pulmonary disease	1.2 (0.6–2.2)
No chronic pulmonary disease	1.6 (1.0–2.3)
PN-panel positive only	1.1 (0.7–1.6)
Chronic pulmonary disease	0.6 (0.3–1.4)
No chronic pulmonary disease	1.3 (0.8–2.2)
Culture positive	1.9 (1.3–2.8)
Chronic pulmonary disease	2.3 (1.2–4.4)
No chronic pulmonary disease	1.8 (1.1–3.0)
Culture positive only	2.6 (1.3–5.3)
Chronic pulmonary disease	2.9 (0.8–10.2)
No chronic pulmonary disease	2.5 (1.0–5.9)
PN-panel and culture positive	1.6 (1.0–2.4)
Chronic pulmonary disease	1.9 (1.0–3.8)
No chronic pulmonary disease	1.5 (0.9–2.6)

In a univariate logistic regression, we found that detection of *H. influenzae, S. pneumoniae*, *S. agalactiae*, and *S. aureus* by the PN-panel was not associated with pneumonia. By contrast, detection of Enterobacterales (composite of *E. coli*, *Proteus* spp.,* Enterobacter cloacae* complex, *K. pneumonia*-group, *K. oxytoca*, and* S. marcescens*) and *P. aeruginosa* in BAL samples was associated with increased risk of pneumonia—however the estimates were based on low numbers, and mainly represented patients in the ICU who had antibiotic prior to sampling (Table S5).

## Discussion

In this study using real-world data, we compared the diagnostic performance of the PN-panel with the culture-based reference approach in BAL samples from hospitalized patients, to evaluate the clinical relevance of the PN-panel for the diagnosis of pneumonia.

Overall, we found that the sensitivity and specificity of the PN-panel was high, whereas the positive predictive value of the detection of a bacterial pathogen was low. The PN-panel detected one or more pathogens in every second patient with pneumonia, however also in almost every second patient without pneumonia, which corresponded with a high prevalence of normal respiratory flora in the samples. Importantly, we found a strong association with pneumonia for BALs that were culture positive only, whereas PN panel positive only was not associated with pneumonia. In our setting MDR is of low prevalence and accordingly the quick detection of resistance markers was of minor clinical relevance.

### Sensitivity

Increasingly, panel PCRs are being implemented in routine clinical microbiology. Indisputably, such tests offer improvements in turnaround time, sensitivity, and accuracy [6]. Overall, sensitivity and specificity of the PN-panel using culture as a gold-standard is high. A large study of 846 BAL and 836 sputum patient samples reported sensitivity higher than 95% for 10 of the bacterial targets, and remaining target where sensitivity could be calculated was between 75 and 92%. Specificity for both specimen types was > 91%. The high performance was also confirmed using real-time PCR and sequencing, which also revealed that false negatives in PN-panel are rare [4]. Nevertheless, non-viable pathogens may be detected in all PCR-based diagnostic methods. One smaller study reported a slightly lower sensitivity for *K. aerogenes* [7], whereas another found that false negatives only occurred, when the bacterial load was lower than < 10^3.5^ [4]. We also found that three *K. aerogenes* detected in culture, were missed in the PN-panel. As *K. aerogenes* is an intrinsic ampC-producing bacterium this could impact on choice of treatment. Other studies performed in the ICU setting, have highlighted that the PN-panel missed important pathogens among their patients including other Enterobacteriaceae (e.g., *Morganella morganii* and *Citrobacter freundii*), enterococci, and aerobic Gram-negative rods (e.g. *S. maltophilia*) [3, 8]. We noted growth of Candida species in many samples. While this is not considered relevant in pneumonia, it may be important in the overall judgment and approach in patients at the intensive care unit (multi-site colonization being a risk factor for invasive fungal infection).

### Clinical relevance

A non-negligible challenge with the use of panel PCRs is the interpretation of the results in respect to the clinical significance. To evaluate the clinical value of such a multiplex PCR, clinical information is required. Importantly, one should also consider “background noise” in form of the normal bacterial flora of the upper respiratory tract which is a mix of commensal microorganisms and potential pathogens. While a BAL should contain only respiratory material from the lower respiratory tract, often commensal flora from upper respiratory tract is also detected. Such “contamination” may lower the sensitivity in culture-based methods due to overgrowth of oral flora with lack of identification of relevant pathogens. Overall, we found that around 40% of patients *without* clinical pneumonia had a positive result in the PN-panel, detecting one or more viral and/or bacterial pathogens. Thus, false positives (detection of microorganisms without clinical importance) in the PN-panel needs consideration. False positive rates have been reported for *S. aureus, H. influenzae*, *M. catarrhalis*, and *P. aeruginosa* [4]. We also found a high detection rate of these bacteria using the PN-panel, whereas it was lower in the culture-based method. A possible explanation could be differences of liquid collected for the respective methods: While 200 μL of BAL is needed for the multiplex PCR, only 1 μL is used for culture—a priori this would favour a higher detection rate using the PN-panel. Also, antibiotic treatment before BAL could have impacted on the higher detection rate by the PN-panel. However, the lower reporting of *S. aureus, H. influenzae*, *M. catarrhalis* in the culture-based methods where growth is evaluated manually, could also reflect an intended non-reporting of these potential pathogens. In our study, the rate of concurrent normal respiratory flora was high (detected using the culture-based method), and likewise in the PN-panel almost a fourth of samples included either *S. aureus* or *H. influenzae*, which were not associated with pneumonia in a univariate logistic regression model. Moreover, when stratifying by underlying chronic pulmonary disease, we found that a positive PN-panel was a predictor of pneumonia only in patients without chronic pulmonary disease, whereas the odds ratio for pneumonia was increased in those with positive culture regardless of underlying chronic pulmonary disease. In a study from Norway, including 72 patients with community-acquired pneumonia, *H. influenzae* and *S. pneumonia* detected by the PN-panel were deemed a relevant cause of the pneumonia. However, among 24 patients with other respiratory tract infections (viral infections), the detection rate of these same bacteria was similar, but deemed not relevant [9]. In a Danish study of 298 patients hospitalized for suspected pneumonia, the clinical sensitivity and specificity of the PN-panel was 70% and 43%, respectively [10]. Even when considering only high-quality sputum samples (evaluated by microscopy of epithelial cells), there was no significant improvement of the performance. The authors pointed out that while efficacy was low for the PN-panel—this also was true for the reference method using culture. Accordingly, the results from the PN-panel needs to be carefully interpreted in combination with the clinical condition, otherwise it could lead to inappropriate use of antibiotics [10]. We found that the PPV increased for several pathogens when restricting to patients with pneumonia, also supporting that the evaluation should be performed considering clinical signs of infection.

A prior study found a strong correlation between genome copies/mL and colony forming units (CFU), as well as higher copy number for pathogens also found in the culture-based method [11]. While we found concordance for 10^4^–10^5^ copies/mL and CFU, copies/mL of 10^6^ or more was not similarly present in culture. Same findings applied after restriction to patients with pneumonia.

### Multidrug resistance

A French multi-center study, which included 515 respiratory specimens analysed using the PN-panel, detected 42 resistance genes [6]. We detected a much lower percentage, which correspond with the low prevalence in our population. Among 259 inpatients tested with PN-panel, a review of patient files showed that antibiotic adjustments could be made in 71% of patients, including discontinuation or de-escalation in 48% of patients [2]. However, this study was performed in a setting with a much higher prevalence of methicillin and carbapenem resistance than in our setting. Thus, the potential for the assay to impact on antibiotic stewardship is likely higher in settings with high resistance problems. Nevertheless, a clinical decision on whether a patient is simply colonized, or the resistant microorganism is the causative pathogen remains regardless of diagnostic modality.

### Important pathogens not included in the PN-panel

In agreement with a prior study on almost 400 samples [11], we also identified growth of *S. maltophilia* and *Achromobacter xylosoxidans—*which may both cause opportunistic pneumonia in compromised patients. Other studies on ICU patients have highlighted the lack of inclusion of *S. maltophilia* in the PN-panel [12, 13]. Moreover, detection of *Aspergillus fumigatus* and other moulds may be highly relevant to detect, to broaden treatment in immunocompromised patients or ICU patients with SARS-CoV-2 infection [14].

### Strengths and limitations

We evaluated the PN-panel using real world data with clinical information. Our study was large compared to the existing literature; we included 840 adult hospitalized patients, covering patients both with and without suspicion of pneumonia (as indications for BAL were either diagnostics of interstitial lung disease or infection). Thus, the samples represented patients without pneumonia (serving as a non-infected comparison group), as well as patients with community- and hospital-acquired pneumonia. All samples came from patients examined at Basel University Hospital, i.e., the sampling and the microbiological examinations were performed using the same standards. However, our study also has several important limitations. We relied on diagnosis from the medical chart based on the treating clinician’s interpretation, rather than a prospective assessment of pneumonia using standardized criteria. BAL is often not possible to perform upfront when a patient is admitted to the hospital, and consequently, a large proportion of patients had antibiotics before sampling. Therefore, the detection rate of culture-based diagnostics was likely reduced, and the distribution of detected pathogens shifted from Gram-positive to more Gram-negative pathogens. As our data derived from a single center, our findings may not be generalizable to other centers with different case mix. Clinical parameters included only the first measurement, which may not be the most relevant measure, and we also lacked important clinical information such as the immune state of the patient. Finally, our results were based on samples from a routine lab, and the impact of the PN-panel on clinical management needs further evaluation in prospective, randomized controlled trials. According to clinicaltrials.gov several trials will examine both use in diagnostics of community-acquired pneumonia, as well as hospital-acquired and ventilator-acquired pneumonia.

Overall, we found the PN-panel did not add substantially to standard culture in pneumonia diagnostics in a clinical setting with low prevalence of multi-drug resistant bacteria. While potential contamination is also an issue in conventional culture, growth by culture is evaluated according to clinical expertise and standards, allowing to separate commensals from true pathogens together with the clinical presentation. These standards must be developed for PCR-based methods. Secondly, while the PN-panel has many targets, it still does not include all relevant pathogens. Among our patients, several cultures revealed opportunistic Gram-negative bacilli such as *S. maltophilia* and *A. xylosoxidans* which are often very resistant, in addition to *A. fumigatus*, which may also be important to consider in the immunocompromised patient.

### Supplementary Information

Below is the link to the electronic supplementary material.

Supplementary file1 (DOCX 38 KB)

## Data Availability

All supportive data are available within the article and its supplementary files.
