# Supplementary material for: Evaluation of the clinical relevance of the Biofire© FilmArray pneumonia panel among hospitalized patients
Source: Infection. 2023 Aug 12;52(1):173–81. doi: 10.1007/s15010-023-02080-1 (PMC10810975; doi:10.1007/s15010-023-02080-1)
Supplement: Supplementary file 1 — Supplementary file1 (DOCX 38 KB) [file 15010_2023_2080_MOESM1_ESM.docx]

**Supplements**

**Supplementary Text**

**Description of microbiological testing**

**Additional findings by the PN-panel**

**Supplementary Tables**

**Table S1. Targets identified by the Biofire FilmArray*^©^* Pneumonia panel.**

**Table S2. Additional microorganisms detected by Biofire FilmArray*^©^* Pneumonia panel and other pathogens or colonizing flora detected by culture (not included in Biofire) in 1078 BALs.**

**Table S3. Semi-quantitative results for 15 targets.** Results in Biofire FilmArray*^©^* Pneumonia panel examined in 1078 bronchoalveolar lavages among patients with and without pneumonia.

**Table S4. Additional microorganisms (not covered by both methods).** List of additional pathogens detected among 175 patients with pneumonia.

**Table S5. Univariate odds ratio of pneumonia among 840 unique hospitalized patients including 175 with pneumonia, according to pathogens detected.**

**Description of microbiological testing**

We obtained microbiological data from the laboratory information system. We analysed all samples within the routine workflow (ISO/IEC 17025 accredited) for species identification of bacteria and molds and performed antibiotic resistance profiling. Presence of normal respiratory flora (e.*g., S. mitis* and other *viridans streptococci,* apathogenic *Neisseria spp.,* Corynebacterium spp., Lactobacillus spp., and Candida spp.) was noted. Briefly, we homogenized BAL samples by vortexing and culturing 1uL of BAL fluid on the following agar plates: Columbia sheep agar (Becton Dickinson GmbH, BD), Haemophilus chocolate agar (bioMérieux), MacConkey agar (BD), and Sabouraud Dextrose agar (BD). Plates were visually inspected for bacterial and fungal growth after 24h and 48h, and for fungal growth with a maximum of six days. Identification of microorganisms was performed using Matrix assisted laser desorption/ionization-time of flight (MALDI-TOF) mass spectrometry (using Bruker Microflex^TM^ or MALDI Biotyper^TM^; Bruker Daltonics) and the MALDI-TOF database (version MBT 8468 MSP Library, BDAL V9.0.0.0_7854-8468). For resistance testing we used single colony isolates of a new subculture and either disk-diffusion or E-Tests (Apteq), or automated microdilution with the VITEK 2 system (bioMérieux), interpreted using EUCAST breakpoints.

*Biofire FilmArray© Pneumonia panel.* The PN-panel includes a total of 35 targets per assay (**Table S1**). We performed the PN-panel according to the manufacturer’s instruction. Briefly, we loaded 200uL of the respiratory sample into the Filmarray cartridge, and the sample was automatically processed and analysed by the Filmarray Torch system (bioMérieux). As the PN-panel was newly implemented and the clinical relevance of findings uncertain, only viral and atypical bacterial pneumonia pathogens, similar to the Biofire Filmarray Respiratory Panel, were reported to the clinicians. In case of detection of an AMR gene, we informed the treating physician.

**Additional findings by the PN-panel**

The PN-panel includes atypical pneumonia pathogens and several viral pathogens (not culturable using standard methods). Among pneumonia patients, the PN-panel detected a few important pathogens requiring specific antibiotic or antiviral treatment, such as *Legionella pneumonia* (n=3) and *Influenza A* (n=2). Among patients without pneumonia, there were no cases positive for *Legionella pneumonia*, but six patients had *Influenza A* detected in the BAL. In addition, several other respiratory viruses were found, including human rhino/enterovirus (n=28), coronavirus (non-SARS-CoV-2; n=6), human metapneumovirus (n=5), parainfluenza virus (n=5), adenovirus (n=3), and respiratory syncytial virus (n=2).

**Table S1. Targets identified by the Biofire FilmArray*^©^* Pneumonia panel.**

| Type |  |
| --- | --- |
| Virus | Adenovirus Coronavirus Human metapneumovirus Human rhinovirus/enterovirus Influenza A virus Influenza B virus Parainfluenza virus Respiratory syncytial virus Middle East respiratory syndrome coronavirus |
| Bacteria | *Acinetobacter calcoaceticus-baumannii* complex *Enterobacter cloacae* complex *Escherichia coli Haemophilus influenzae Klebsiella aerogenes Klebsiella oxytoca Klebsiella pneumoniae* group *Moraxella catarrhalis Proteus* spp. *Pseudomonas aeruginosa Serratia marcescens Staphylococcus aureus Streptococcus agalactiae Streptococcus pneumoniae Streptococcus pyogenes* |
| Atypical bacteria | *Chlamydia pneumoniae Mycoplasma pneumoniae*  *Legionella pneumoniae* |
| Antimicrobial resistance genes | *mecA*/*mecC* and MREJ^a^ KPC*^b^*  NDM*^b^* OXA-48-like^c^ VIM*^b^* IMP*^b^* CTX-M*^b^* |

^a^Reported when *S. aureus* is detected. *^b^*Reported when *A. calcoaceticus*-*baumannii* complex, *E. cloacae* complex, *E. coli*, *K. aerogenes*, *K. oxytoca*, *K. pneumoniae* group, *Proteus* spp., *P. aeruginosa*, or *S. marcescens* is detected. *c*Reported when *E. cloacae* complex, *E. coli*, *K. aerogenes*, *K. oxytoca*, *K. pneumoniae* group, *Proteus* spp., or *S. marcescens* is detected.

**Table S2. Additional microorganisms detected by Biofire FilmArray*^©^* Pneumonia panel (not examined for using culture) and other pathogens or colonizing flora detected by culture (not included in PN panel) in 1078 BALs.**

| **Additional pathogens Biofire panel** | **N** |
| --- | --- |
| *Mycoplasma pneumoniae* | 3 |
| *Legionella pneumoniae* | 3 |
| *Adenovirus* | 4 |
| *Human Metapneumovirus* | 10 |
| *Human Rhino/Enterovirus* | 94 |
| *Coronavirus* | 16 |
| *Influenza A* | 8 |
| *Parainfluenza Virus* | 17 |
| RSV | 14 |
| **Other pathogens or colonizing flora detected in culture** | **N** |
| Enterococci | 10 |
| Viridans streptococci | 2 |
| Coagulase-negative staphylococci and Corynebacterium spp. | 6 |
| Neisseria spp. and pasteurella spp. | 4 |
| *Achromobacter xylosoxidans* | 2 |
| *Burkholderia cepacia-complex* | 2 |
| *Enterobacteriacea (*including *Klebsiella variicola, Citrobacter freundii, C. koseri, Morganella morganii, Hafnia alvei)* | 16 |
| *Stenotrophomonas maltophilia* | 4 |
| Gram-negative rods (unspecified) | 16 |
| Anaerobes | 2 |
| *Candida* species (including *C. albicans* (n=44), *C. glabrata* (n=11), C*. parapsilosis* (n=1), C*. guilliermondii* (n=1), *C. tropicalis* (n=2) *Candida spp*. (n=21) | 80 |
| *Aspergillus fumigatus* | 7 |
| *Aspergillus species (non-fumigatus)* | 1 |
| *Fusarium species* | 1 |
| *Penicillium species* | 5 |
| *Geotrichum species* | 2 |
| *Rhizomucor species* | 1 |
| *Rhizopus species* | 1 |
| *Trichoderma species* | 1 |
| Mould (not further specified) | 8 |
| Normal flora | 848 |

**Table S3. Semi-quantitative results for 15 targets.** Results in Biofire FilmArray*^©^* Pneumonia panel examined in 1078 bronchoalveolar lavages among patients with and without pneumonia.

|  | **Pneumonia (232 BALs from 175 patients)** | | | | | | | | | **No pneumonia (846 BAL among 665 patients)** | | | | | | | | |
| --- | --- | --- | --- | --- | --- | --- | --- | --- | --- | --- | --- | --- | --- | --- | --- | --- | --- | --- |
|  | **Biofire Genome copies/mL** | | | | | **Culture Colony forming units/mL** | | | | **Biofire Genome copies/mL** | | | | | **Culture Colony forming units/mL** | | | |
|  | **All** | **10^4^** | **10^5^** | **10^6^** | **>=10^7^** | **All** | **<=10^4^** | **10^4^-<=10^5^** | **>10^5^** | **All** | **10^4^** | **10^5^** | **10^6^** | **>=10^7^** | **All** | **<=10^4^** | **10^4^-<=10^5^** | **>10^5^** |
| **Species, n (%)** |  |  |  |  |  |  |  |  |  |  |  |  |  |  |  |  |  |  |
| *Acinetobacter calcoaceticus-baumannii* complex | 1 | 1 (100) | 0 | 0 | 0 | 0 | 0 | 0 | 0 | 2 | 2 (100) | 0 | 0 | 0 | 0 | 0 | 0 | 0 |
| *Enterobacter cloacae* complex | 5 | 2 (40) | 2 (40) | 1 (20) | 0 | 4 | 1 (25) | 3 (75) | 0 | 11 | 7 (64) | 2 (18) | 0 | 2 (18) | 4 | 0 | 4 (100) | 0 |
| *Escherichia coli* | 12 | 3 (25) | 6 (50) | 1 (8) | 2 (16) | 9 | 3 (33) | 6 (67) | 0 | 31 | 18 (58) | 7 (23) | 1 (3) | 5 (16) | 12 | 7 (58) | 5 (42) | 0 |
| *Haemophilus influenzae* | 26 | 9 (35) | 9 (35) | 3 (12) | 5 (19) | 6 | 1 (17) | 5 (83) | 0 | 117 | 47 (40) | 34 (29) | 21 (18) | 15 (13) | 11 | 0 | 11 (100) | 0 |
| *Klebsiella aerogenes* | 1 | 0 | 1 (100) | 0 | 0 | 2 | 0 | 2 (100) | 0 | 0 | 0 | 0 | 0 | 0 | 0 | 0 | 0 | 0 |
| *Klebsiella oxytoca* | 0 | 0 | 0 | 0 | 0 | 0 | 0 | 0 | 0 | 3 | 2 (67) | 1 (33) | 0 | 0 | 1 | 0 | 1 (100) | 0 |
| *Klebsiella pneumoniae* group | 4 | 2 (50) | 1 (25) | 0 | 1 (25) | 2 | 1 (50) | 1 (50) | 0 | 15 | 7 (47) | 7 (47) | 1 (6) | 0 | 8 | 3 (37) | 5 (63) | 0 |
| *Moraxella catarrhalis* | 5 | 2 (40) | 0 | 1 (20) | 2 (40) | 1 | 0 | 1 (100) | 0 | 23 | 9 (39) | 11 (48) | 1 (4) | 2 (9) | 3 | 0 | 3 (100) | 0 |
| *Proteus* spp. | 6 | 2 (33) | 3 (50) | 1 (17) | 0 | 2 | 0 | 2 (100) | 0 | 5 | 0 | 2 (40) | 3 (60) | 0 | 5 | 2 (40) | 3 (60) | 0 |
| *Pseudomonas aeruginosa* | 21 | 0 | 5 (33) | 9 (38) | 6 (29) | 14 | 3 (21) | 10 (71) | 1 (8) | 33 | 5 (15) | 10 (30) | 9 (27) | 10 (30) | 25 | 6 (24) | 19 (76) | 0 |
| *Serratia marcescens* | 2 | 2 (100) | 0 | 0 | 0 | 1 | 0 | 1 (100) | 0 | 7 | 6 (86) | 1 (14) | 0 | 0 | 2 | 1 (33) | 1 (67) | 0 |
| *Staphylococcus aureus* | 19 | 7 (37) | 7 (37) | 3 (11) | 3 (16) | 11 | 4 (36) | 7 (64) | 0 | 79 | 40 (51) | 14 (18) | 15 (19) | 9 (11) | 37 | 14 (38) | 22 (59) | 1 (3) |
| *Streptococcus agalactiae* | 5 | 3 (60) | 2 (40) | 0 | 0 | 1 | 0 | 1 (100) | 0 | 14 | 8 (57) | 4 (29) | 0 | 2 (14) | 1 | 0 | 1 (100) | 0 |
| *Streptococcus pneumoniae* | 14 | 6 (43) | 3 (21) | 3 (21) | 2 (14) | 5 | 3 (60) | 2 (40) | 0 | 40 | 21 (53) | 13 (32) | 2 (5) | 4 (10) | 18 | 6 (33) | 12 (67) | 0 |
| *Streptococcus pyogenes* | 2 | 0 | 0 | 1 (50) | 1 (50) | 0 | 0 | 0 | 0 | 3 | 2 (67) | 1 (33) | 0 | 0 | 0 | 0 | 0 | 0 |
| **Total, n (%)** | 126 (100) | 37 (29) | 40 (32) | 23 (18) | 24 (19) | 58 (100) | 16 (28) | 41 (71) | 1 (1) | 380 (100) | 176 (46) | 106 (28) | 53 (14) | 49 (13) | 127 (100) | 39 (31) | 87 (69) | 1 (<1) |

**Table S4. Additional microorganisms (not covered by both methods).** List of additional pathogens detected among 175 patients with pneumonia.

|  | **PN +** | **Culture +** |
| --- | --- | --- |
| **Atypical pneumonia pathogens** |  |  |
| *Chlamydia pneumoniae* | 0 | - |
| *Mycoplasma pneumoniae* | 3 | - |
| *Legionella pneumonia* | 3 | 0 |
| **Viral pathogens** |  |  |
| *Adenovirus* | 3 | - |
| *Coronavirus* | 6 | - |
| *Human metapneumovirus* | 5 | - |
| *Human rhino/enterovirus* | 28 | - |
| *Influenza A* | 2 | - |
| *Influenza B* | 0 | - |
| *Parainfluenza virus* | 5 | - |
| *Respiratory syncytial virus* | 2 | - |
| *Middle East respiratory syndrome coronavirus* | 0 | - |
| **Other bacterial pathogens or colonizing flora** |  |  |
| *Streptococcus pseudopneumoniae* | - | 1 |
| *Streptococcus anginosus-group* | - | 1 |
| *Staphylococcus haemolyticus* | - | 1 |
| *Enterococcus species* (*E. faecalis,* (n=1), *E. faecium* (n=4), *E. avium* (n=1)) | - | 6 |
| *Rothia mucilaginosa* | - | 1 |
| *Lactobacillus* | - | 1 |
| *Neisseria meningitidis* | - | 1 |
| *Enterobacteriacea (*including *Klebsiella variicola* (n=1), *Citrobacter koseri* (n=2), *Hafnia alvei (n=1)* and Gram-negative rods (not further specified) (n=3) | - | 7 |
| *Stenotrophomonas maltophilia* | - | 4 |
| ESBL-producing enterobacterales (*K. pneumoniae* (n=1), *E. coli* (n=1)) | 2 | 0* |
| *Actinomyces odontolyticus* | - | 1 |
| Anaerobic bacteria (not further identified) | - | 1 |
| **Fungal pathogens or colonizing flora** |  |  |
| *Aspergillus fumigatus* | - | 4 |
| *Geotrichum* candidum | - | 1 |
| Mould (not further specified) | - | 5 |
| *Candida albicans* | - | 23 |
| *Candida spp. (non-albicans or not specified)* | - | 16 |
| **Normal flora** | - | 168 |

* *E. coli* was detected in culture in low amount, but susceptibility testing was not performed

**Table S5. Odds ratio for bacterial pneumoniae among 840 unique hospitalized patients including 175 with pneumonia, according to pathogens detected**.

|  | **Biofire FilmArray*^©^* Pneumonia panel** | | |
| --- | --- | --- | --- |
| **Bacterial species** | **Samples**  **(n)** | **Pneumonia**  **(n)** | **Odds ratio**  **(95% CI)** |
| ***S. aureus*** | 75 | 12 | 0.7 (0.4-1.3) |
| ***S. agalactiae*** | 18 | 5 | 1.5 (0.5-4.2) |
| ***S. pneumoniae*** | 49 | 13 | 1.4 (0.7-2.7) |
| ***H. influenzae*** | 125 | 25 | 0.9 (0.6-1.5) |
| **Enterobacterales group*** | 66 | 20 | 1.7 (1.0-3.0) |
| ***P. aeruginosa*** | 31 | 13 | 2.9 (1.4-6.0) |

* Enterobacterales group combined *E. coli, Proteus spp., Enterobacter cloacae* complex, *K. pneumonia*-group, *K. oxytoca, and S. marcescens.*
